# The carboxy terminal coiled-coil modulates Orai1 internalization during meiosis

**DOI:** 10.1038/s41598-021-82048-z

**Published:** 2021-01-27

**Authors:** Rawad Hodeify, Maya Dib, Ethel Alcantara-Adap, Raphael Courjaret, Nancy Nader, Cleo Z. Reyes, Ayat S. Hammad, Satanay Hubrack, Fang Yu, Khaled Machaca

**Affiliations:** 1 Department of Physiology and Biophysics, Ca2+ signaling Group, Weill Cornell Medicine Qatar, Education City, Qatar Foundation, Doha, Qatar; 2 College of Health and Life Sciences, Hamad Bin Khalifa University, Doha, Qatar; 3 Present Address: Department of Biotechnology, American University of Ras Al Khaimah, Ras al Khaimah, UAE; 4 Present Address: Lehigh Valley Health Network, Allentown, PA USA; 5 Present Address: Sidra Medicine, Doha, Qatar

**Keywords:** Calcium channels, Endocytosis

## Abstract

Regulation of Ca^2+^ signaling is critical for the progression of cell division, especially during meiosis to prepare the egg for fertilization. The primary Ca^2+^ influx pathway in oocytes is Store-Operated Ca^2+^ Entry (SOCE). SOCE is tightly regulated during meiosis, including internalization of the SOCE channel, Orai1. Orai1 is a four-pass membrane protein with cytosolic N- and C-termini. Orai1 internalization requires a caveolin binding motif (CBM) in the N-terminus as well as the C-terminal cytosolic domain. However, the molecular determinant for Orai1 endocytosis in the C-terminus are not known. Here we show that the Orai1 C-terminus modulates Orai1 endocytosis during meiosis through a structural motif that is based on the strength of the C-terminal intersubunit coiled coil (CC) domains. Deletion mutants show that a minimal C-terminal sequence after transmembrane domain 4 (residues 260–275) supports Orai1 internalization. We refer to this region as the C-terminus Internalization Handle (CIH). Access to CIH however is dependent on the strength of the intersubunit CC. Mutants that increase the stability of the coiled coil prevent internalization independent of specific mutation. We further used human and *Xenopus* Orai isoforms with different propensity to form C-terminal CC and show a strong correlation between the strength of the CC and Orai internalization. Furthermore, Orai1 internalization does not depend on clathrin, flotillin or PIP2. Collectively these results argue that Orai1 internalization requires both the N-terminal CBM and C-terminal CIH where access to CIH is controlled by the strength of intersubunit C-terminal CC.

## Introduction

Store-operated calcium entry (SOCE) is a predominant Ca^2+^ influx pathway in non-excitable cells that regulates cellular homeostasis and physiology^1^. Upon store depletion the ER Ca^2+^ sensor STIM1 clusters and localizes to ER-PM Contact Sites (ER-PM CS), where it directly binds to Orai1, which is a highly selective Ca^2+^ channel at the plasma membrane (PM)^1^. Orai1 is a four-pass transmembrane protein with N- and C- termini facing the cytosol^1^.

The crystal structure of Orai1 reveals a hexameric channel with antiparallel intersubunit coiled coils at the periphery of the channel, formed by a C-terminal domain (residues 263–285)^2,3^. Previous studies showed that both the N- and C- termini are important for Orai1 gating, despite the lower binding affinity of the N-terminus to STIM1^4–6^. In addition to their role in channel activation, the N- and C-termini regulate the residence of Orai1 at the PM. The number of PM Orai1 channels is an important determinant of SOCE levels and is increased in response to store depletion through a STIM1-dependent trafficking trap^7–9^. Furthermore, the intracellular loop between TM2 and TM3 also regulates Orai1 membrane residence through interactions with the CCT chaperonin complex^7^.

Orai1 trafficking plays an important role in shaping Ca^2+^ signals at fertilization, since Orai1 is internalized during *Xenopus* oocyte meiosis through a caveolin-, Rab5-, and dynamin-dependent endocytic pathway^9^. This internalization requires a caveolin binding motif (CBM) in the Orai1 N-terminus (residues 52–60)^9^. Furthermore, deleting the C-terminal 22 residues of Orai1 (1–266 deletion) prevents Orai1 internalization during meiosis^9^. However, the specific molecular determinant within the Orai1 C-terminus required for its internalization are not known.

Herein, we dissect the role of the Orai1 cytosolic C-terminus in its internalization during meiosis. We show a requirement for a short C-terminal sequence right after transmembrane domain 4 (TM4) consisting of residues 260–275 for Orai1 internalization. We refer to this region as the C-terminus Internalization Handle (CIH). We further show that the strength of the C-terminal intersubunit coiled-coil regulates Orai1 endocytosis presumably by controlling access to CIH.

## Results

### The C-terminus of Orai1 is required for its internalization

**Figure 1:**
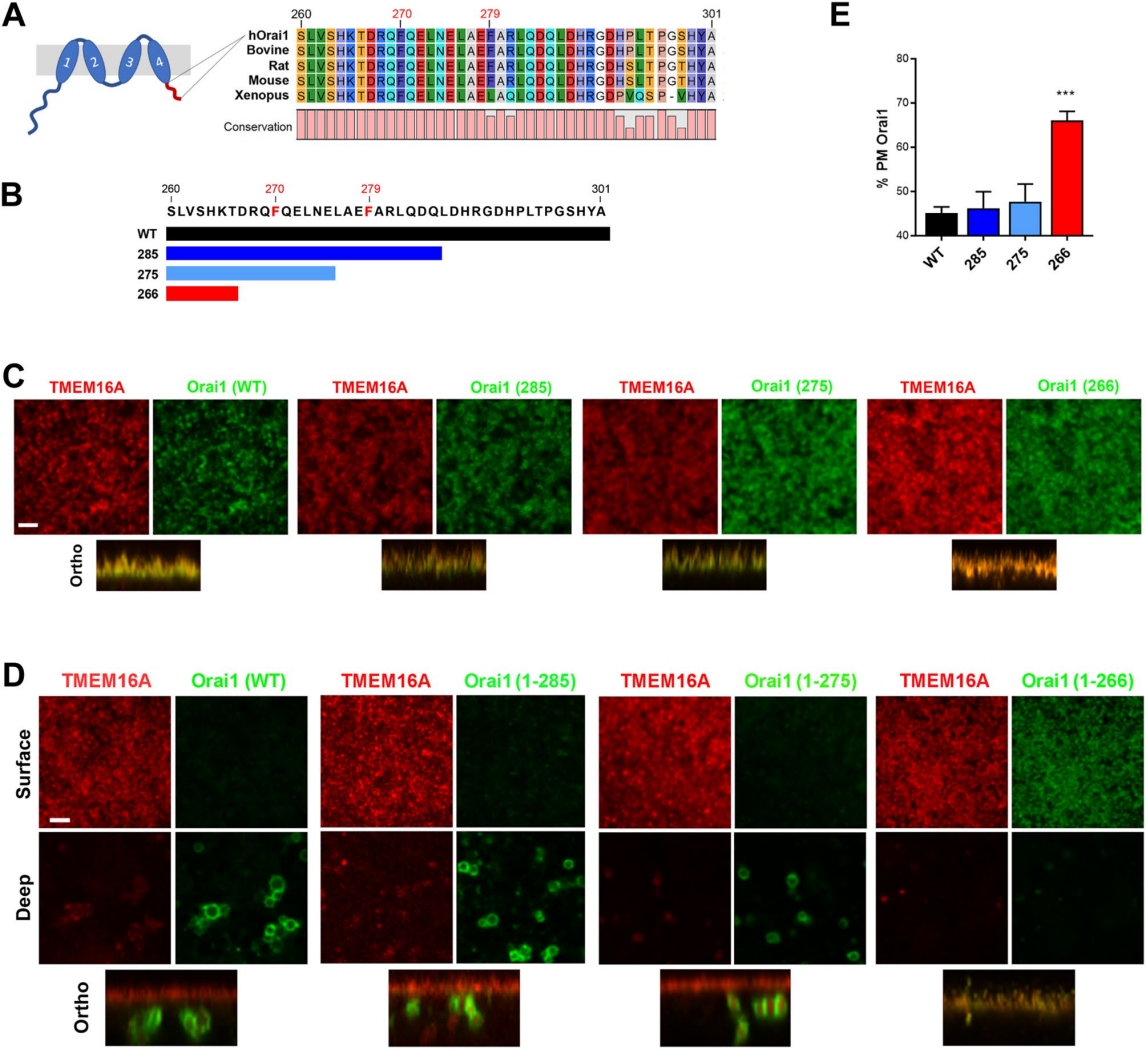
Internalization of Orai1 C-terminal truncations. (**A**) Cartoon of Orai1 with the C-terminus cytoplasmic domain highlighted (red) with its corresponding sequence alignment from various vertebrates. Human (NP_116179), bovine (NP_001092472), mouse (NP_780632), rat (NP_001014004), *Xenopus* (Q5EAU0). (**B**) Representation of the different Orai1 C-terminal deletions. (**C**, **D**) Confocal images and orthogonal sections from *Xenopus* oocytes (**C**) and eggs (**D**) expressing TMEM16A-mCherry with GFP-tagged Orai1 wild-type (WT) or the different deletions (10 ng RNA/oocyte for 48 h) as indicated. Cells were imaged through the PM generating a z-stack of images. Surface indicates the PM focal plane and deep the cytoplasm. Ortho shows an orthogonal cross section across the entire z-stack. Scale is 3 µm. (**E**) Quantification of the percent Orai1 at the PM as described in Methods (Mean ± SEM, n = 7–29 eggs from 6 donor females). *** (p < 0.001), one-way ANOVA.

Orai1 is composed of 301 residues with a short cytoplasmic C-terminus (residues 260–301) that is highly conserved among vertebrates (Fig. 1A). We previously showed that a mutant Orai1 with residues 267–301 deleted (Orai1 1–266) traffics normally in oocytes (arrested at the G2/M transition of the cell cycle) but in contrast to wild-type Orai1 is not internalized during meiosis^9^ (Fig. 1). This argues that the Orai1 C-terminus contains molecular determinants required for targeting it for endocytosis during meiosis. To define these, we generated three nested Orai1 deletions 1–266, 1–275 and 1–285 (Fig. 1B). All three deletions traffic normally to the PM in oocytes and co-localize with the Ca^2+^-activated Cl channel Ano1 (TMEM16A), a marker for the PM^9^, in a similar fashion to full-length Orai1 (Fig. 1C). During meiosis Orai1 (WT), and the 1–275 and 1–285 deletions are endocytosed into an intracellular vesicular pool (Fig. 1D,E). In contrast, the Orai1(1–266) deletion is retained at the PM in eggs (Fig. 1D,E). We quantified Orai1 internalization by measuring the percentage of Orai1 localizing to the PM in confocal z-stacks, with the PM marked by TMEM16A (Fig. 1E). These data suggest that the C-terminal cytoplasmic region between residues 260–275, which follows immediately after transmembrane domain 4 (TM4) forming the entry into the cytosol (Fig. 1A), is critical for efficient Orai1 internalization during meiosis. We refer to this domain as the C-terminus Internalization Handle (CIH).

We used the human Orai1 (hOrai1) clone for these studies as it is the most extensively characterized in terms of its trafficking during oocyte meiosis^9,10^. The *Xenopus* Orai1 replicates the trafficking of hOrai1 as it is internalized into an intracellular vesicular pool during meiosis (Supp. Figure 1).

### Mutating the FXXΦ motifs in the Orai1 C-terminus prevents Orai1 internalization

**Figure 2:**
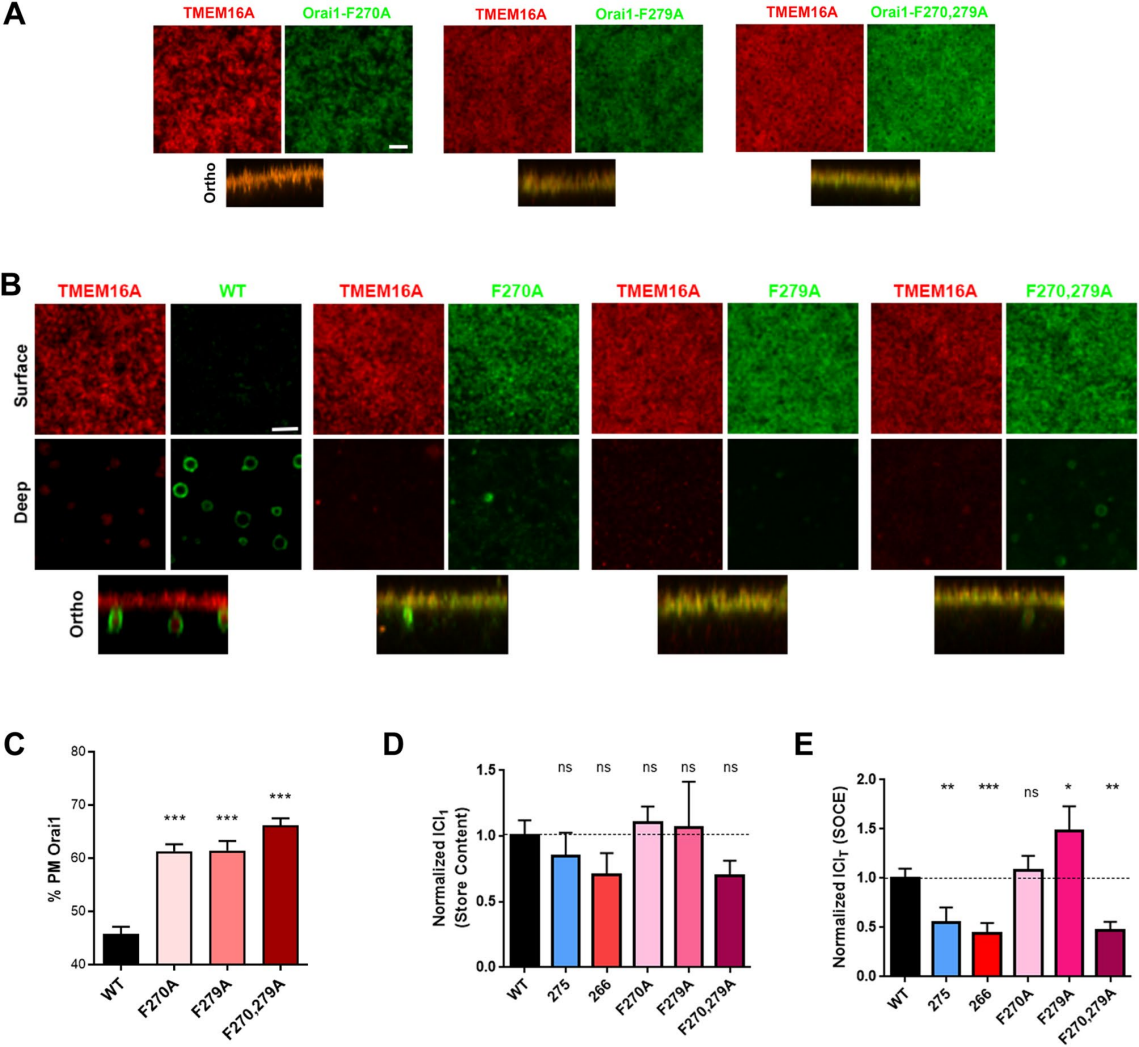
Internalization and functionality of the FXXΦ mutants. (**A**, **B**) Confocal images from oocytes (**A**) and eggs (**B**) expressing TMEM16A with GFP-Orai1 WT, F270A, F279A, or F270,279A mutants under similar conditions as in Fig. 1C and 1D. Scale bar is 3 µm. (**C**) Percent Orai1 at the PM (Mean ± SEM, n = 12–42 eggs from 9 donor females). (**C**, **D**) Normalized Cl_1_ (I_Cl1_) (**C**) and Cl_T_ (I_ClT_) (**D**) currents from oocytes expressing STIM1 with the different Orai1 mutants as indicated (Mean ± SEM, n = 5–25 oocytes from 5 donor females). *(p < 0.05), **(p < 0.01), *** (p < 0.001), ns (not significant), one-way ANOVA.

The Orai1 C-terminus contains two stretches, 270-FQEL-274 and 279-FARL-282 (Fig. 1A), that match the tyrosine-based motif consensus sequence for protein internalization YXXΦ, where Y could be replaced with F, and Φ represents a hydrophobic residue^11^. These motifs partially overlap with the 266–275 region identified as critical for Orai1 internalization in the deletion analyses (Fig. 1A). We therefore mutated F270, F279, or both residues F270,279 to Ala, and tested their internalization during meiosis. All three mutants traffic express and traffic normally to the PM in oocytes (Fig. 2A). In contrast, Orai1 internalization during meiosis was inhibited in all three mutants (Fig. 2B,C).

To test whether either the deletions or point mutations alter Orai1 function, we co-expressed each mutant with STIM1 in oocytes, and measured Ca^2+^-activated Cl currents in response to store depletion, which reflects Ca^2+^ entry through SOCE^12,13^. The ability of CaCCs to differentiate between and faithfully report Ca^2+^ release from stores and Ca^2+^ influx through SOCE have been extensively characterized previously^12–16^. Details of the CaCCs measurements with example traces for each mutant are shown in Supplemental Fig. 2. I_Cl1_ responds to Ca^2+^ release from stores (Supp. Figure 2A) and is not significantly affected by expression of any Orai1 mutant (Fig. 2D and Supp. Figure 2B). In contrast, I_ClT_ faithfully reports Ca^2+^ influx through SOCE (Supp. Figure 2A) and is significantly inhibited in oocytes expressing the 1–275 and 1–266 deletions as well as the F270,279A mutant (Fig. 2E and Supp. Figure 2C). The Orai1 C-terminus has been shown to be critical for STIM1 interaction with a requirement for coiled coil formation^4,5,17^. Consistently, we observe inhibition of SOCE in the 1–275 and 1–266 mutants as they remove portions of the C-terminus required for STIM1 interaction. As shown above none of the mutants affect Orai1 trafficking to the PM in oocytes, so the inhibition of SOCE in F270,279A is not due to defective PM targeting. The reason underlying SOCE inhibition in F270,279A was not clear initially, although later experiments (see Fig. 4) offer an explanation.

These results argue that there is no correlation between the ability of the different Orai1 mutants to interact with STIM1 and their internalization during meiosis. The F270A and F279A mutants form functional channels that interact with STIM1 as they are gated normally by store depletion when expressed with STIM1 and support Ca^2+^ influx as indicated by similar levels of CaCCs as compared to WT Orai1 (Fig. 2E). Yet these mutants are not internalized during meiosis (Fig. 2C). In contrast the 1–275 deletion is internalized during meiosis (Fig. 1E), yet it does not support SOCE. Finally, the double mutant F270,279A and the 266 deletion are neither internalized (Fig. 1E and 2C) nor do they support Ca^2+^ influx (Fig. 2E).

### The internalization of F270, F279 mutants is not rescued by Rab5 or caveolin1

**Figure 3:**
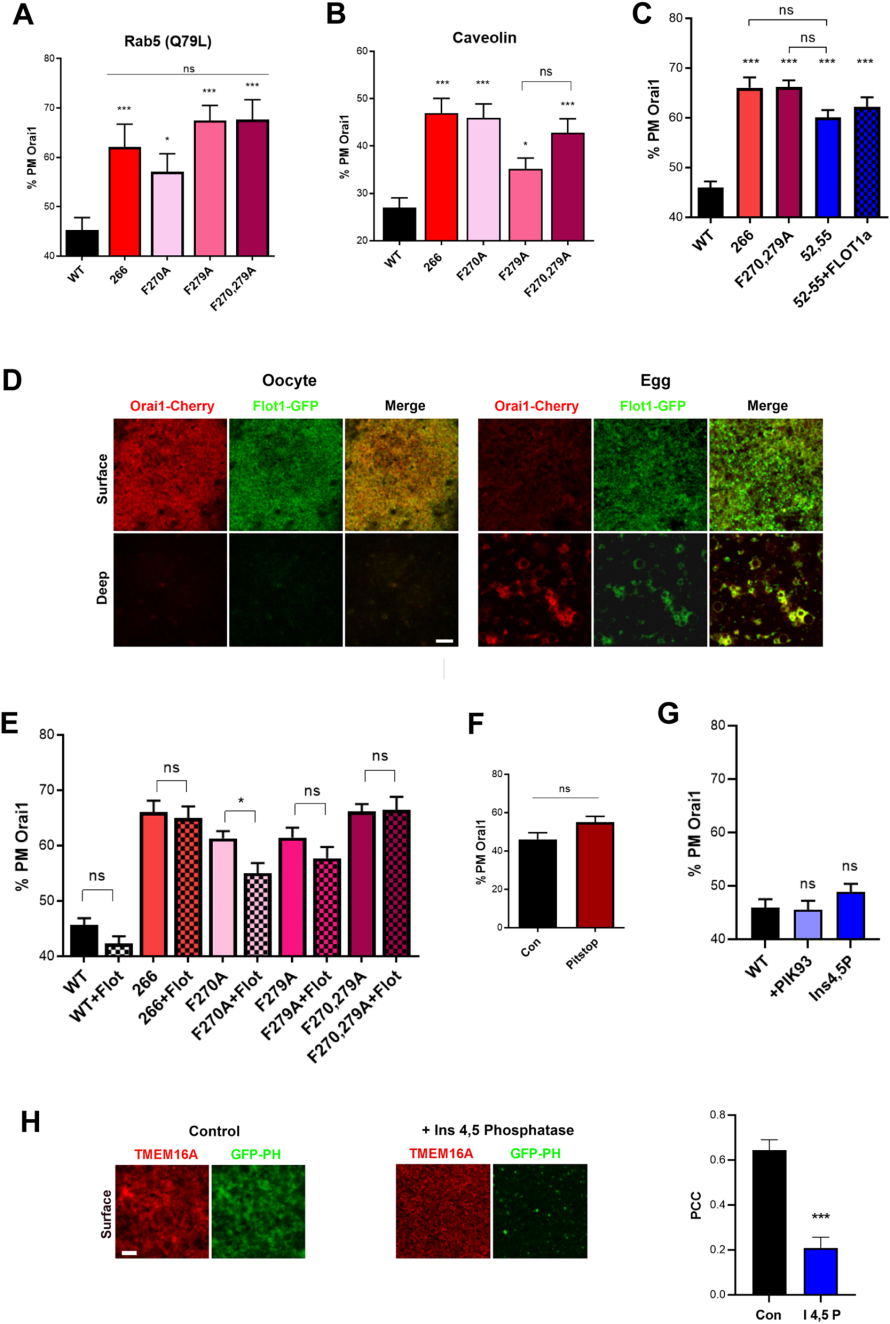
Role of flotillin, PIP2 and clathrin in Orai1 internalization. (**A**, **B**) Quantification of the percent Orai1 at the PM in eggs expressing the different mutants as indicated with either a constitutively active Rab5 mutant (Q79L) (**A**) or caveolin (**B**) (Mean ± SEM, for (**A**) n = 10–26 eggs, for (**B**) n = 7–23 eggs). *(p < 0.05), *** (p < 0.001), ns (not significant) , one-way ANOVA. (**C**) Percent Orai1 at the PM for the different conditions as indicated (Mean ± SEM, n = 12–19 eggs from 4 donor females. *** (p < 0.001), ns (not significant), one-way ANOVA. (**D**) Confocal images from oocytes and eggs expressing mCherry-Orai1 and flotillin1-GFP (Flot1-GFP). Scale bar is 3 µm. (**E**) Percent Orai1 at the PM for the different conditions as indicated (Mean ± SEM, n = 11–57 eggs from 5 donor females). * (p < 0.05), ns (not significant), one-way ANOVA. (**F**) Percent PM Orai1 in eggs pretreated with either vehicle (DMSO) or Pitstop 2 (10^–5^ M) 2 h after progesterone addition to block clathrin-dependent endocytosis (Mean ± SEM, n = 10–13 eggs from 3 donor females), ns (not significant), unpaired t test. (**G**) Percent PM Orai1 in eggs treated with PIK93 (0.5 µM) or expressing Ins4,5P (Mean ± SEM, n = 15–28 eggs from 3 donor females). ns (not significant), one-way ANOVA. (**H**) Confocal images from eggs co-expressing GFP-PH and TMEM16A-mCherry (Control) or co-injected with Ins4,5P RNA (+ Ins 4,5 Phosphate). Scale bar is 3 µm. The right panel shows the Pearson Correlation Coefficient for TMEM16A and GFP-PH at the PM plane (Mean ± SEM, n = 7 eggs). *** (p < 0.001), unpaired t test.

Previous work showed that Orai1 internalization is caveolin- and Rab5-dependent^9^. We therefore overexpressed the constitutively active Rab5 mutant (Q79L) but did not observe any rescue of either the 1–266, F270A, F279A, or F270,279A mutants during meiosis (Fig. 3A). We further show that expression of caveolin with the different mutants does not improve their internalization during meiosis (Fig. 3B). This despite the fact that Orai1 internalization depends on the caveolin binding motif (CBM) in the Orai1 N-terminus^9^. Mutating CBM (52-YPDWIGQSY-60) to Y52,W55A inhibits Orai1 internalization during meiosis to a similar extent as the 266 deletion and the F270,279A mutant (Fig. 3C).

### Orai1 internalization is not dependent on flotillin or clathrin

In search of endocytic pathways other than caveolin that may contribute to Orai1 internalization through the F270 and F279 motifs, we tested the role of flotillin. Flotillins are integral membrane proteins that are abundant in lipid rafts and are involved in endocytosis. We co-expressed Cherry-Orai1 and flotillin1-GFP (Flot-1-GFP) in oocytes and eggs. In oocytes, Orai1 co-localizes with Flot-1 at the PM, and in eggs the two proteins co-localize in intracellular vesicular compartments (Fig. 3D). In *Xenopus*, flotillin-1 exists in two isoforms 1a and 1b^18^. Flotillin-1a or 1b do not rescue Orai1 internalization when expressed with the 266 deletion, the caveolin Y52,W55A mutant (52–55) (Fig. 3C), the F279A, or the F270,279A mutants (Fig. 3E). Flotillin expression increased F270A internalization but did not reach similar levels of internalization as WT Orai1 (Fig. 3E). These results suggest that flotillin does not play a major role in mediating Orai1 internalization.

We further tested whether inhibiting clathrin-dependent endocytosis blocks Orai1 internalization. Using transferrin as a marker for clathrin-dependent endocytosis we previously showed that the clathrin-dependent endocytosis inhibitor Pitstop2 (10^–5^ M) effectively blocks transferrin uptake (Supp. Figure 3A)^19^, however it did not interfere with Orai1 internalization (Fig. 3F). Therefore, Orai1 does not appear to be taken up through the clathrin-dependent pathway during meiosis but rather relies primarily on caveolin-dependent endocytosis.

### Phosphatidylinositol (4,5)-bisphosphate (PIP2) is not involved in Orai1 internalization

Several studies reveal an important role for PIP2 in membrane trafficking and PM-cytoskeleton rearrangement during endocytosis. PIP2 was shown to bind adaptor proteins necessary for coat formation^20^, and cytoskeletal proteins involved in actin organization, including cofilin-1, profilin, and annexin^21–23^. Thus, PIP2 serves as an assembly hub for actin-adaptor proteins during endocytosis. We were therefore interested in testing whether PIP2 regulates Orai1 internalization.

PIP2 levels are determined by the balance between synthesis by phosphatidylinositol 4-kinase and phosphatidylinositol 4-phosphate 5-kinase and hydrolysis by phosphatidylinositol 4,5-bisphosphate phosphatase or by phospholipase C. PIP2 is generated by lipid kinases in two sequential phosphorylation steps from PtdIns and PtdIns(4)P. The first step is catalyzed by PI4Ks, mainly PI4KIIIα. It was previously reported that depletion of either of PI4KIIα, PI4KIIIα, or PI4KIIIβ caused a ∼25% decrease in PIP2 levels^24^. PIK-93 is a dual PI3K and PI4K inhibitor, with highest potency against PI4IIIKβ^24^. We treated oocytes with PIK-93 (0.5 µM) or vehicle (DMSO) before stimulating meiosis and did not observe any effect on Orai1 internalization (Fig. 3G).

However, PIK-93 at the concentrations used may not effectively inhibit the oocyte enzymes, we therefore tested whether depletion of PIP2 by expressing the catalytic Ins 4,5 Phosphatase (Ins4,5P) affects Orai1 internalization. To evaluate the efficacy of Ins4,5P, we co-expressed the GFP-tagged PIP2 reporter (GFP-PH). Using TMEM16A as a surface marker, we show that Ins4,5P expression depletes PIP2 at the PM as shown by loss of GFP-PH signal at the surface and decreased colocalization between the TMEM-mCherry and GFP-PH signals (Fig. 3H). This however did not affect the internalization of Orai1 (Fig. 3G). We further confirmed that PIP2 removal from the PM was effective throughout the oocyte maturation time course as it was observed in both oocytes and mature eggs (Supp. Figure 3B). Ins 4,5 Phosphatase was associated with translocation of GFP-PH to intracellularly (Supp. Figure 3B). This could be due to binding to other inositol phosphate species or the inability of Ins4,5P to hydrolyze intracellular PIP2 leading to its enrichment. These data argue that PM PIP2 does not modulate Orai1 internalization during meiosis.

### Orai1 C-terminal coiled-coil stability defines its internalization

Collectively the above results argue against a role for clathrin, PIP2 or flotillin-dependent endocytic pathways in Orai1 internalization and point to caveolin-dependent endocytosis as the sole pathway. This argues that somehow the caveolin-endocytic machinery interacts with both the N- and C-termini of Orai1. There is precedent for this type of interaction for Orai1 as the STIM1 CAD/SOAR domain has been shown to interact with both the N- and C-terminus of Orai1^4,5,17,25^.

**Figure 4:**
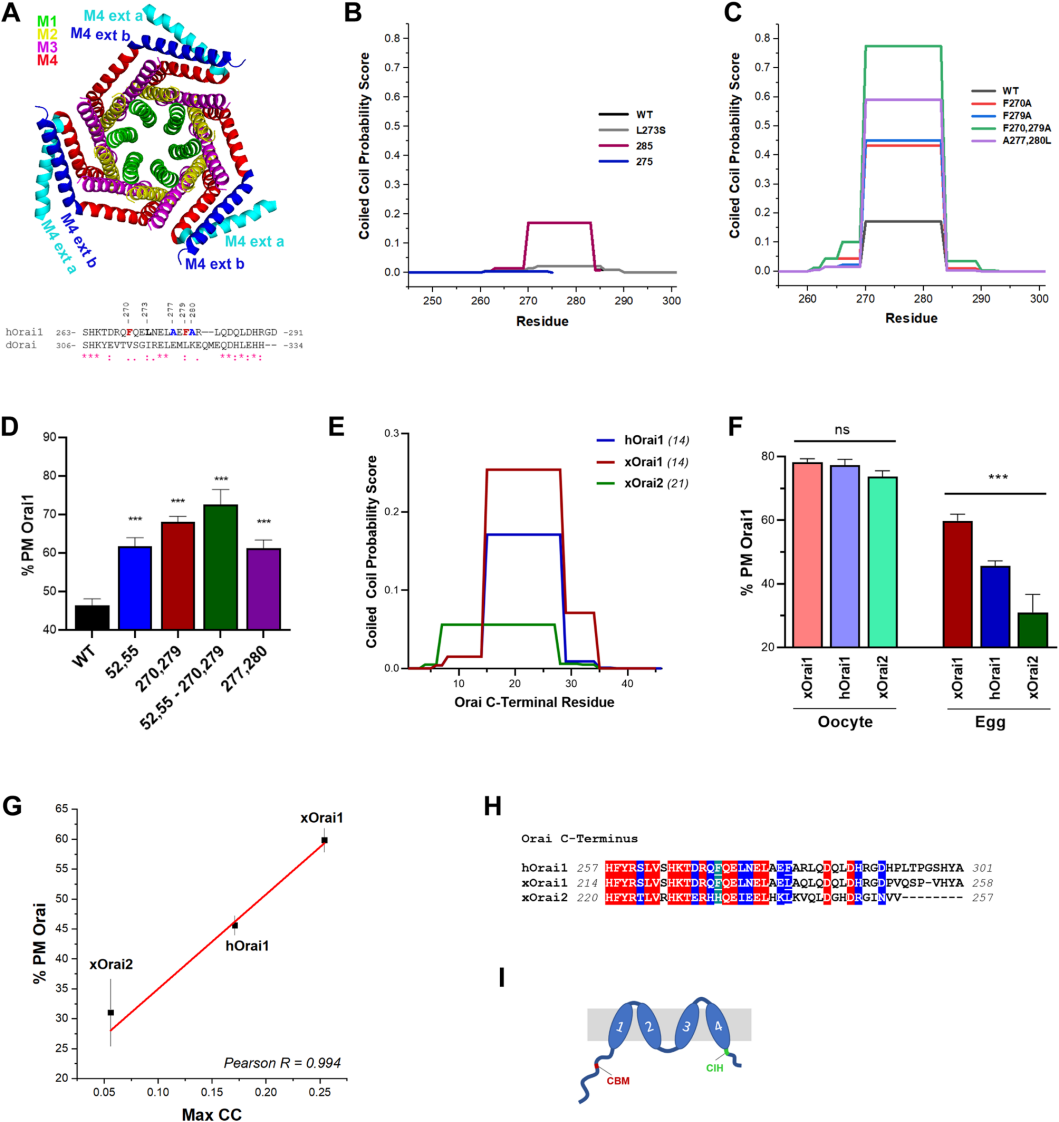
C-terminal CC structure regulates Orai1 internalization. (**A**) Orai1 crystal structure in the closed conformation (4HKR) with the 4 transmembrane helices in the hexameric Orai1 channel color coded as indicated. The M4 helical extensions that form the CC from adjacent subunits are shown in dark and light blue. CC sequence alignment between human and *Drosophila* Orai1 is also shown. (**B**, **C**) CC predictions using the COILS algorithm with a window of 14 residues^41^. (**D**) Percent PM Orai1 for the different mutants as indicated (Mean ± SEM, n = 13–26 eggs from 4 donor females). *** (p < 0.001), one way ANOVA. (**E**) CC predictions for the different Orai isoforms using the COILS algorithm. In this case the window that gave the highest CC probability is shown and is indicated in italics in parentheses. Human Orai1 (hOrai1, Q96D31), *Xenopus* Orai1 (xOrai1, Q5EAU0), and *Xenopus* Orai2 (xOrai2, Q6NZI6). (**F**) Percent Orai at the PM in both oocytes and eggs for the different clones as in panel (E). Mean ± SEM, n = 9–27 eggs from 3 donor females. *** (p < 0.001), ns (not significant), one way ANOVA. (**G**) Plot of percent Orai at the PM as a function of maximal CC probability for the three Orai isoforms. (**H**) Sequence alignment of the C-termini of hOrai1, xOrai1 and xOrai2. (**I**) Cartoon topology of Orai1 showing the CBM domain in the N-terminal cytosolic region and cytoplasmic internalization handle domain (CIH) in the Orai C-terminus.

A domain within the Orai1 C-terminus forms anti-parallel coiled coils between adjacent subunits that has been referred to as the M4-extension (Fig. 4A), because it forms a continuous α-helix in the open configuration of the channel^2,3^. Furthermore, the Orai1 C-terminal domain including its ability to form a coiled coil (CC) are critical for interaction with STIM1 to gate Orai1^4,5^. Indeed, coiled coil algorithms predict a CC between residues 269–285 (Fig. 4B). The L273S mutation is predicted to disrupt the CC (Fig. 4B) as previously shown^5^. We had previously shown that the L273S mutant does not traffic properly to the PM in oocytes, so it is not possible to test its internalization during meiosis^9^. The 285 Orai1 deletion retains the CC (Fig. 4B), whereas the 275 Orai1 deletion is not predicted to form a CC (Fig. 4B), yet both deletions are internalized during meiosis (Fig. 1E). In contrast, the 266 deletion is missing the majority of the cytoplasmic C-terminus including the CC and is not internalized during meiosis (Fig. 1E). This argues for an important role of the CC in Orai1 endocytosis during meiosis.

Based on these findings we considered the possibility that the FXXϕ motifs in the Orai1 C-terminus may not be acting as internalization motifs, but rather that mutating these residues may be affecting C-terminal CC formation. Two lines of evidence support this line of thinking: (1) the 275 deletion is missing the F279XXϕ motif and internalizes during meiosis (Fig. 1E), arguing that F279XXϕ is dispensable for internalization. In contrast, the single F279A mutant does not internalize (Fig. 2A,B), arguing that F279XXϕ is essential for internalization if it is acting as an internalization motif. (2) the lack of effect of the endocytic pathways tested (other than caveolin) on Orai1 internalization.

Consistent with this logic the F270A, F279A and the double mutant lead to significant stabilization of the CC in the Orai1 C-terminus (Fig. 4C). So, these mutations may be affecting Orai1 internalization by stabilizing the CC, rather than acting as direct internalization motifs. If that is the case it stands to argue that other mutants that increase CC stability without affecting the FXXϕ motifs will be defective in their internalization during meiosis. We designed a double mutant at residues A277 and A280 within human Orai1 [which are not conserved with Drosophila Orai1 (Fig. 4A)] to leucine. The A277,280L mutant is predicted to significantly stabilize the CC to similar levels as those observed in the F270,279A mutants (Fig. 4C). Interestingly, the A277,280L mutant is not internalized during meiosis, and shows a similar retention at the PM as is observed with the caveolin (Y52,W55A) or the F270,279A mutants (Fig. 4D). Besides, when we combine the Y52,W55,F270,F279A mutations, we observe a higher retention of Orai1 at the PM (Fig. 4D). These data argue that the stability of the CC in the Orai1 C-terminus regulates Orai1 internalization during meiosis, rather than any sequence-specific internalization motif.

To further test this conclusion, we assessed the internalization during meiosis of human Orai1 (hOrai1), *Xenopus* Orai1 (xOrai1) and *Xenopus* Orai2 (xOrai2) as their C-termini are predicted to have differing CC probabilities (Fig. 4E). The CC probability of xOrai1 is the highest with that of hOrai1 being intermediate and xOrai2 the lowest (Fig. 4E). The gradual decrease in CC probability in these three Orai isoforms offers a test for the correlation between CC probability and Orai endocytosis during meiosis. Furthermore, conveniently all three Orai isoforms show a CC probability lower than that of the mutants that are not internalized during meiosis (below 0.4, see Fig. 4C). All three Orai isoforms traffic efficiently to the PM in oocytes and show similar levels of enrichment at the PM (Fig. 4F). In contrast, during meiosis xOrai1 is the least efficiently internalized followed by hOrai1 and then xOrai2 showing the most efficient internalization (Fig. 4F). The levels of Orai at the PM in eggs is statistically different among the three isoforms as well as for each isoform as compared to the oocyte control (p < 0.001) (Fig. 4F). We plotted Orai internalization as a function of CC probability for the three Orai isoforms to assess their correlation (Fig. 4G). The Pearson’s correlation of 0.994 argues for a strong dependence of Orai internalization on the C-terminal CC probability (Fig. 4G). Of note, sequence alignment of the three isoforms C-termini shows that F279 is not conserved whereas F270 is weakly conserved (Fig. 4H), again supporting the conclusion that these Tyr-based motifs are not involved in Orai1 internalization during meiosis. Importantly though and despite these isoform-specific sequence variations in the C-terminus, the caveolin binding motif (CBM) is fully conserved in all three isoforms (Supp. Figure 4A).

## Discussion

Collectively our data reveal that the probability of CC formation in the Orai1 C-terminus regulates its trafficking during meiosis. This is the case whether we introduce different mutations in Orai1 that alter CC probability or whether we use different Orai isoforms with varying C-terminal CC probabilities (Fig. 4). As the probability for the Orai C-terminal to form a CC increases this is strongly associated with gradual inhibition of Orai1 internalization.

In addition to the low CC probability there is also a requirement for a minimal cytosolic C-terminal sequence to support Orai1 internalization. The 266 deletion is not internalized despite the fact that it is not predicted to form a CC. The 275 deletion is also predicted not to form a CC yet internalizes to similar levels as WT Orai1 (Fig. 1E). This argues that the Orai1 C-terminus needs to extend into the cytosol by a minimal length to support internalization. This is defined here by the 275 deletion as a region between residues 260 and 275, assuming that TM4 exits the bilayer at residue 261 based on prediction algorithms and the Orai1 crystal structure. We refer to this domain as the C-terminal internalization handle (CIH) (Fig. 4I), as it is needed for internalization without any defined endocytic sequence motifs. Based on these results we propose a model where Orai1 endocytosis during meiosis requires interaction of the caveolin endocytic machinery with the N-terminal CBM domain, as well as a bridging interaction with the Orai CIH in the C-terminus (Fig. 4I) to effectively support Orai1 endocytosis and PM removal during meiosis. Access to the C-terminal CIH domain in full-length Orai1 requires a loosely structured CC. If the probability of intersubunit CCs in the Orai C-terminus is high this results in poor endocytosis (see Fig. 4). We speculate that as CC formation probability increases this limits access of the endocytic machinery to CIH and as such decrease Orai internalization during meiosis.

The model we propose to regulate Orai1 internalization during meiosis requires interaction of the endocytic machinery with both the N- and C-termini of Orai1. Significant work has established similar interactions between STIM1 and both Orai1 termini that regulate multiple aspects of SOCE function including Orai1 clustering into puncta, gating and permeation^4,5,17,25^. STIM1 binds the Orai1 C-terminus robustly and this binding requires a propensity to form the intersubunit CC^4,5,17^. In addition, STIM1 binds to the Orai1 N-terminus and although this binding is weaker than to the C-terminus, it possibly modulates channel activation^4^. There is also evidence that STIM1 binding to the Orai1 C-terminus is sufficient for gating and that binding of STIM1 to the N-terminal is dispensable^26^. This is supported by studies using constitutively active Orai mutants suggesting that the N-terminus, particularly the so-called extended transmembrane Orai N-terminal (ETON) region, is important for Orai gating and permeation independent of STIM1 binding^3,4,26–29^.

The caveolin binding motif (CBM) localizes to residues 52–60 in the Orai1 N-terminus and is as such distinct but in close proximity to the presumed STIM1 binding region/ETON, which localizes to residues 73–90 (Supp. Figure 4A). So, one could imagine steric hinderance between STIM1 and caveolin binding. It is worth noting that there are questions as to whether the CBM is sufficient for interaction with caveolin because of its broad distribution and because it is often imbedded inside some proteins questioning its accessibility^30^. Nonetheless, for Orai1 it is clear that mutating the CBM prevents its internalization^9^.

In contrast, there is overlap between the STIM1 binding region in the Orai1 C-terminus and the CIH domain required for Orai1 endocytosis during meiosis. We show here that the higher the probability of CC formation in the Orai1 C-terminus the less efficiently it is endocytosed during meiosis (Fig. 4), presumably due to more limited access of the caveolin endocytic machinery to the CIH region. However, Orai1 endocytosis does not require CC formation in the C-terminus since the 275 deletion does not form CC yet is internalized to similar levels as wild-type Orai1 (Fig. 1E). In contrast, STIM1 binding requires the propensity to form a CC^4,5^. Therefore, the propensity to form a CC in the Orai1 C-terminus regulates both Orai1 gating, clustering and permeation as well as its internalization during meiosis, but in opposite directions.

In that context, the C-termini of the three human Orai isoforms Orai1, 2 and 3 have significantly different probabilities to form a CC with Orai3 having the strongest probability and Orai1 the lowest (Supp. Figure 4B). Given our current knowledge regarding STIM1 interaction with the Orai1 C-terminal CC and the data presented in this paper it is tempting to speculate that the probability of CC formation modulates the function of the different Orai isoforms. There is indeed evidence for this for the different Orais. Both Orai2 and Orai3 are missing three residues (EFA, see Supp Fig. 4C) in their C-termini compared to Orai1. Removing this region in Orai1 (277–279 deletion) increases Orai1 CC probability and improves its interaction with STIM1 as shown convincingly using FRET and SOCE current measurements^31^. Furthermore, addition of the missing EFA to the Orai3 C-terminus, which is predicted to decrease CC probability, results in increased current^32^.

Of note, Orai1 internalization appears to be specific to oocyte meiosis^9,10,33^ and is not observed during mitosis^34^. Furthermore, during interphase Orai1 recycles continuously with rapid kinetics at the PM and its membrane residence is modulated by Ca^2+^ store content as well interaction with the CCT chaperonin complex^7,8^. There could be important developmental underpinnings for Orai1 internalization in oocyte meiosis as the early embryo needs to form a polarized epithelium to support formation of a fluid-filled cavity during gastrulation^35^. This requires vectorial fluid transport that is supported during oocyte maturation by internalization of many ionic channels and transporters (including Orai1) that will contribute to the formation of the basal membrane of this epithelium, while the PM of the oocytes forms the apical layer thus allowing vectorial salt and water transport^35^.

## Methods

### Materials

Wild *Xenopus laevis* female frogs were ordered from Xenopus Express (Le Bourg, France). Tricaine (Ethyl 3-aminobenzoate methanesulfonate), L15 media (L4386) from Sigma. Collagenase IA from (Affymetrix), PIK-93 from Selleckchem (Germany).

### Molecular biology

*Xenopus laevis* flotillin1a-HA, flotillin1a-GFP, flotillin1b-HA, and flotillin1b-GFP, in pCS107^18,36^ were gifts from Ira Daar (National Cancer Institute, Maryland). The pCR3-GFP-Inp54p and pCR3-Inp54p plasmids were kindly provided by Anthony Lai (Cardiff University)^37^. The GFP-C1-PLCdelta-PH was from Addgene (plasmid # 21,179)^38^. The pSGEM-GFP-PH plasmid was constructed by subcloning GFP-PLCdelta-PH into SpeI-EcoRV of pSGEM after PCR amplification using the following primers: (5′-TAAGCTAGTATGGTGAGCAAGGGCGAGGAGCTG-3′; 5′-ACGCCGCTTGATATCTTACTGGATGTTGAGCTCCTT-3′). GFP-myc-Orai1 and mCherry-STIM1 constructs^39^ were subcloned into pSGEM as described previously^9^. *Xenopus* expression plasmids pSGEM-mCherry-xCav1 and pSGEM-mRFP-Rab5 were described previously^9^. To construct pSGEM-mCherry-Myc-Orai1, Myc-Orai1 fragment was amplified by PCR using pSGEM-GFP-myc-Orai1 as template and sub-cloned into XhoI-BamHI sites of pmCherry-N1 plasmid using the primer pair: 5′- CAGATCTCGAGCCATGGAGCAAAAGCTCATTTCTGAG-3′; 5′-GTGAAGGATCCCTAGGCATAGTGGCTG-3′. The resulting vector was used as template to amplify mCherry-Myc-Orai1 fragment using the primers: 5′-CAAGGATCCACATGGAGCAAAAGCTCATTTCTGAG-3′; 5′-GTGAACTCGAGCTAGGCATAGTGGCTG-3′. mCherry-Myc-Orai1 fragment was inserted into BamHI-XhoI sites of the pSGEM vector**.**

To construct pSGEM-GFP-myc-Orai1(1–266), pSGEM-GFP-myc-Orai1(1–275), and pSGEM-GFP-myc-Orai1(1–285), nucleotides at respective positions in the template vector pSGEM-GFP-myc-Orai1 were substituted to generate premature stop codon using the Quickchange mutagenesis kit (Stratagene). Similarly, pSGEM-GFP-myc-Orai1-F270A, pSGEM-GFP-myc-Orai1-F279A, and pSGEM-GFP-myc-Orai1-F270AF279A point mutants were generated by substitution of phenylalanine at respective positions to alanine, while pSGEM-GFP-myc-Orai1A277,280L mutant was generated by substitution of alanines to leucines. The Y52,W55,F270,F279A mutant was generated by substitution of tyrosine and tryptophan to alanine in the F270,279A mutant. All mutants and constructs were verified by DNA sequencing and by analytical endonuclease restriction enzyme digestion.

The primers used are:

- Orai1 (1–266).
- Forward: 5′- GCTCACTGGTTAGCCATAAGACTTAGCGACAGTTCCAG -3′.
- Reverse: 5′- CTGGAACTGTCGCTAAGTCTTATGGCTAACCAGTGAGC -3′.
- Orai1 (1–275).
- Forward: 5′- CAGGAGCTCAACGAGTAGGCGGAGTTTGCCCG -3′.
- Reverse: 5′- CGGGCAAACTCCGCCTACTCGTTGAGCTCCTG -3′.
- Orai1(1–285).
- Forward: 5′- TCCCCTCTGTGGTCCTACTGGTCCTGTAAGCG -3′.
- Reverse: 5′- CGCTTACAGGACCAGTAGGACCACAGAGGGGA -3′.
- Orai1-F270A.
- Forward: 5′- CATAAGACTGACCGACAGGCCCAGGAGCTCAACGAGC -3′.
- Reverse: 5′- GCTCGTTGAGCTCCTGGGCCTGTCGGTCAGTCTTATG -3′.
- Orai1-F279A.
- 5′- CAACGAGCTGGCGGAGGCTGCCCGCTTACAGGAC -3’
- 5′- GTCCTGTAAGCGGGCAGCCTCCGCCAGCTCGTTG -3’
- Orai1-A277,280L.
- 5′- GCTCAACGAGCTGCTGGAGTTTCTCCGCTTACAGGACCAGCTGG -3’5′- GCTCAACGAGCTGCTGGAGTTTCTCCGCTTACAGGACCAGCTGG -3’
- 5′- CCAGCTGGTCCTGTAAGCGGAGAAACTCCAGCAGCTCGTTGAGC -3’
- Orai1 Y52A, W55A.
- 5′- CGTCCGCCGTCACCGCCCCGGACGCGATCGGCCAGAGTTACTC -3’
- 5′- GAGTAACTCTGGCCGATCGCGTCCGGGGCGGTGACGGCGGACG -3’

### Xenopus oocyte expression

To generate RNAs plasmids were linearized with NheI or PvuI for GFP-Inp54p and -Inp54p clones, or Asp718 for the flotillin clones and RNAs synthesized by in vitro transcription using the mMessage mMachine T7 kit (Ambion).

### Oocyte isolation and preparation

Animals were handled according to Weill Cornell Medicine College Institutional Animal Care and Use Committee (IACUC) approved procedures (protocol #2011–0035). Stage VI *Xenopus* oocytes were obtained as previously described^16,40^. Oocytes were injected with RNA and kept at 18 °C for 1–2 days after injection to allow for protein expression. Oocyte maturation was induced with 5 μg/ml progesterone. Germinal vesicle breakdown (GVBD), which is indicative of meiosis progression, was detected visually by the appearance of a white spot at the animal pole.

### Confocal imaging and analysis

Confocal imaging was performed live on either a Leica (Leica, Germany) or a Ziess LSM880 confocal fitted with a Plan Apo 63x/1.4 oil immersion objective. Z-stacks of 0.5 um sections using a 1 Airy unit pinhole aperture were performed across the plasma membrane. Typically, scans are done on a 512 × 512 pixels frame using an averaging of 2 scans per channel. Images were analyzed using LAS AF (Leica) and ImageJ software (http://imagej.net/mbf/). To quantify Orai1 mutants subcellular distribution in eggs and oocytes, the peak of TMEM16A-mCherry fluorescence (max fluorescence) was used to mark plasma membrane; the intensity of fluorescence was analyzed through a z-stack of images, where we conservatively used the peak of TMEM16A-mCherry fluorescence to mark the plasma membrane. The sum of GFP intensity of the stacks from first z-slice moving inward to 1 z-slice below the peak TMEM16A-mCherry was considered surface. The sum intensity of GFP fluorescence of the slices below the peak are considered cytoplasm. For rescue experiments with Rab5, the peak signal of Rab5-mCherry was considered to be cytoplasmic. For rescue experiments with caveolin1-mCherry, the sum of GFP intensity of z slices above caveolin1-mCherry peak was considered PM.

### Statistical analysis

Values are given as means ± SEM. Statistical analysis was performed using student unpaired t-test and one-way ANOVA as indicated with p values indicated as follows: *(p < 0.05), **(p < 0.01), *** (p < 0.001), and ns (not significant). Statistics were obtained using Prism 7 or 8.3 (Graphpad Software, La Jolla, USA).

## Supplementary Information

Supplementary information.
