# Supplementary material for: The carboxy terminal coiled-coil modulates Orai1 internalization during meiosis
Source: Sci Rep. 2021 Jan 27;11:2290. doi: 10.1038/s41598-021-82048-z (PMC7840751; doi:10.1038/s41598-021-82048-z)

The carboxy terminal coiled-coil modulates Orai1 internalization during meiosis

Rawad Hodeify, Maya Dib, Ethel Alcantara-Adap, Raphael Courjaret, Nancy Nader, Cleo Z Reyes, Ayat S. Hammad, Satanay Hubrack, Fang Yu and Khaled Machaca

Supplemental Data

Supplemental Figure Legends

Supplemental Figure 1. ***Xenopus*** **Orai1 is internalized during oocyte meiosis.**

A. Example confocal images at the plasma membrane plane (Surface) or in a cytosolic plane (Deep) and orthogonal sections from *Xenopus* oocytes and mature eggs expressing TMEM16A-mCherry with GFP-tagged *Xenopus* Orai1 (xOrai1). Oocytes were imaged through the PM generating a z-stack of images. Ortho shows an orthogonal cross section across the entire z-stack to show the distribution of TMEM and Orai1. Scale is 5 µm. **B.** Quantification of the percent xOrai1 at the PM in both oocytes and eggs. Mean + SEM, n = 16-27 eggs from 3 different donor females. *** ($p<0.001$), unpaired t test.

Supplemental Figure 2. Ca^{2+}-activated chloride currents (CaCC) to quantify Ca^{2+} release and SOCE.

A. Cartoon of Ca^{2+} release and Ca^{2+} influx activation of CaCC and example traces of activation of I_{Cl1} in response to Ca^{2+} release from store (top traces) as well as I_{ClT} in response for Ca^{2+} influx through SOCE following store depletion. Ionomycin (Ion) was used to deplete stores in this case. The voltage protocol used is shown above the traces and was designed to depolarize the cell membrane using the +40 mV pulse to minimize the driving force for Ca^{2+} influx, with the -140 mV pulse designed to induce Ca^{2+} influx following store depletion to measure SOCE. SOCE was measured using the transient Cl current during the second +40 mV pulse (IClT) that responds to Ca^{2+} influx during the -140 mV pulse [1]. **B-C.** Example traces from the different mutants during the Ca^{2+} release phase (I_{Cl1}) from the first +40 mV pulse (B) and SOCE phase (I_{ClT}) from the second +40 mV pulse (C).

Supplemental Figure 3.

A. Quantification of intracellular transferrin fluorescence intensity normalized to the control treatment for oocytes treated with vehicle control (Veh.), Pitstop2 and its control (10^{-5} M) (mean $\pm$ SEM; 15 oocytes per condition, from 3 donor females). *** ($p<0.001$), one way ANOVA. These data are reproduced here from Nader et al. 2020 [2]. **B.** Representative confocal images and orthogonal cross sections across the z-stack for oocytes and eggs injected with GFP-PH to assess the levels of PIP2 with or without Ins 4,5 phosphatase (+ Ins 4,5 Ptase).

Supplemental Figure 4.

A. Sequence alignment of the N-termini of Human Orai1 (hOrai1, Q96D31), *Xenopus* Orai1 (xOrai1, Q5EAU0), and *Xenopus* Orai2 (xOrai2, Q6NZI6). The caveolin binding motif with its consensus is indicated as well as the extended transmembrane Orai N-terminal (ETON) of Orai1. **B.** CC predictions for the different human Orai isoforms using the COILS algorithm. The window with the highest CC probability is shown and is indicated in italics in parentheses. hOrai1 (Q96D31), hOrai2 (Q96SN7), hOrai3 (Q9BRQ5). **C.** Sequence alignment of the N-termini of Human Orai isoforms.

References

1 Courjaret, R. & Machaca, K. Xenopus Oocyte As a Model System to Study Store-Operated Ca(2+) Entry (SOCE). *Front Cell Dev Biol* **4**, 66, doi:10.3389/fcell.2016.00066 (2016).

2 Nader, N. *et al.* Membrane progesterone receptor induces meiosis in Xenopus oocytes through endocytosis into signaling endosomes and interaction with APPL1 and Akt2. *PLoS Biol.* **18**, e3000901, doi:10.1371/journal.pbio.3000901 (2020).

Supplementat Figure 1

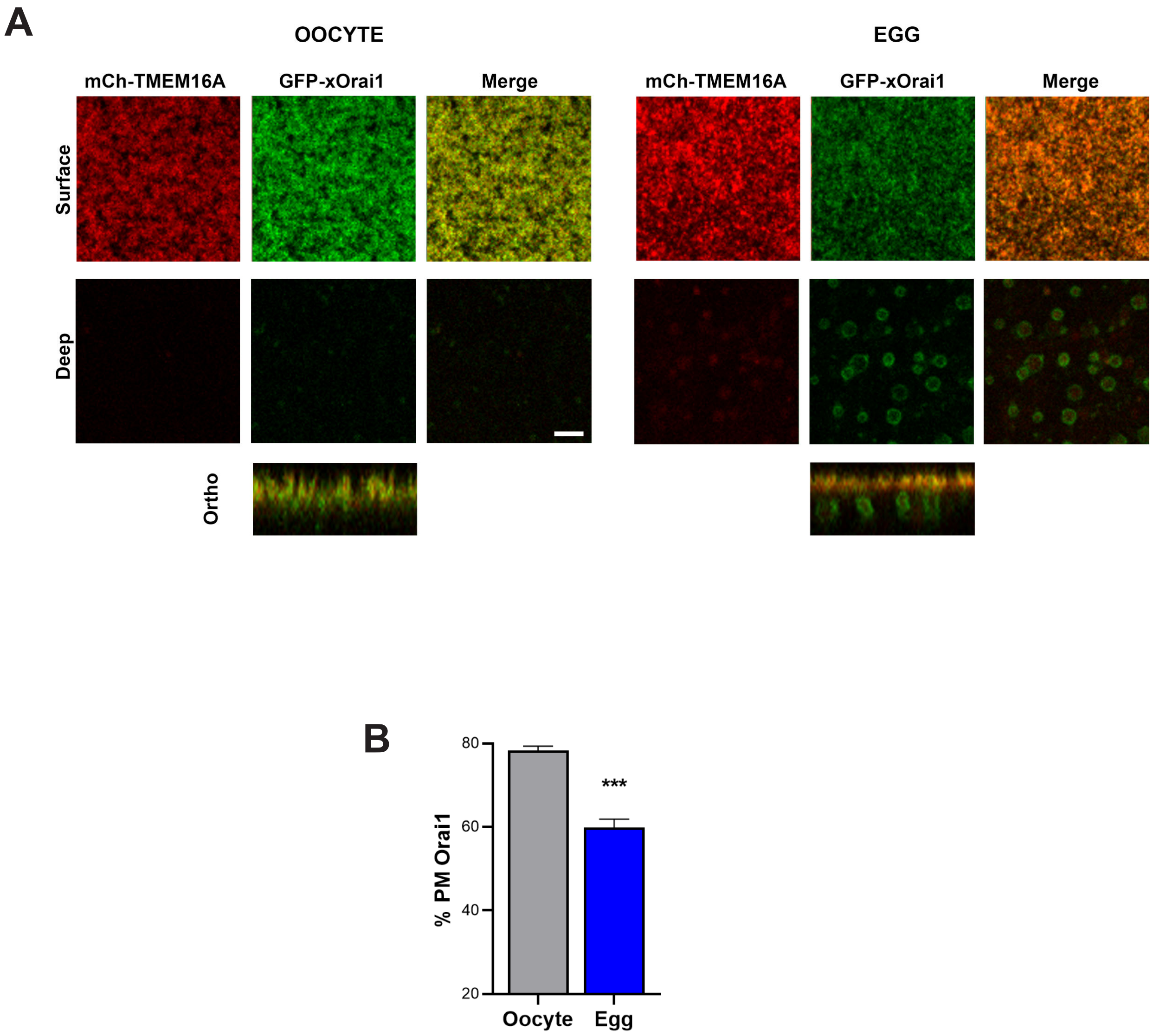

Supplementat Figure 2

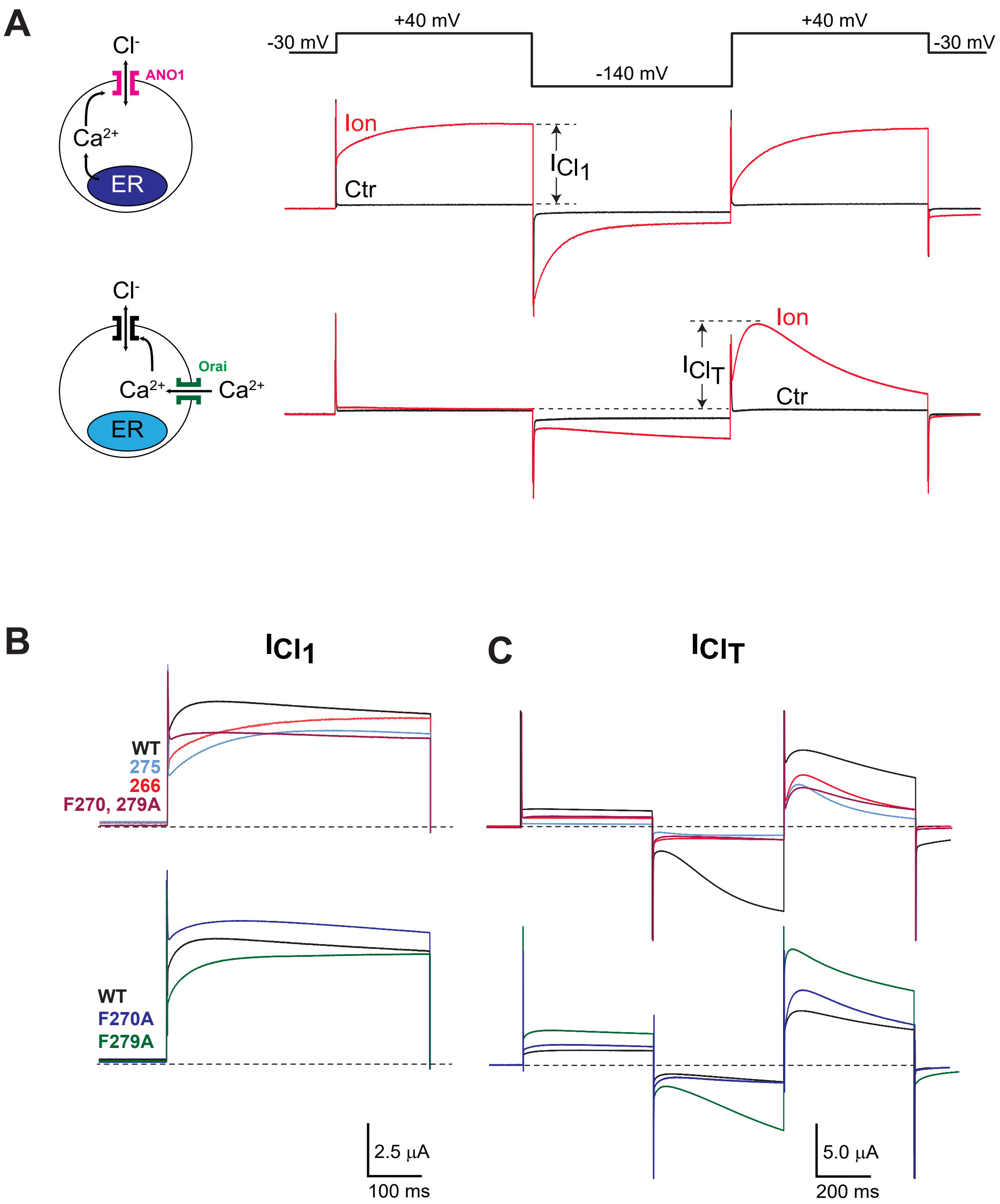

Supplementat Figure 3

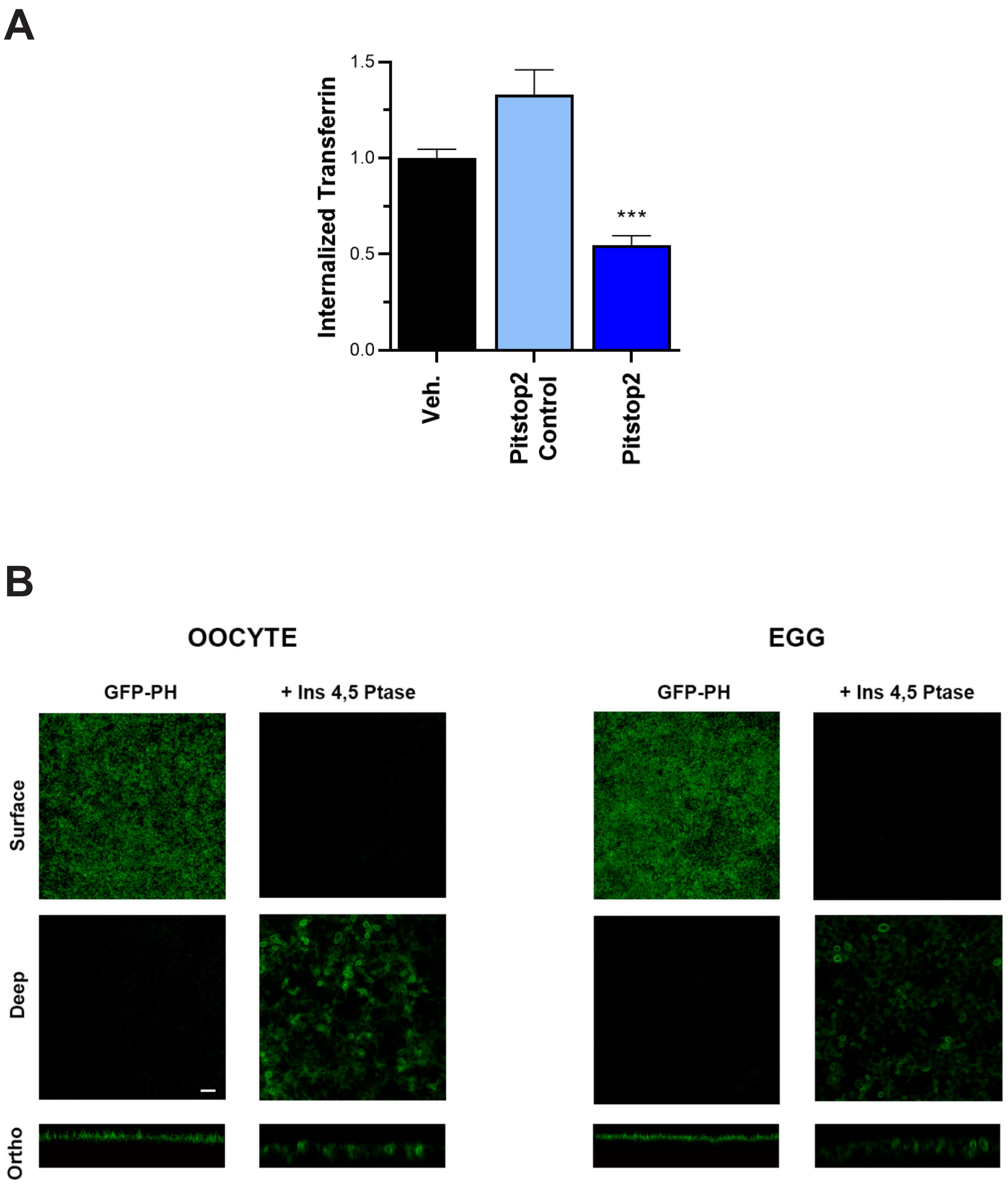

Supplementat Figure 4

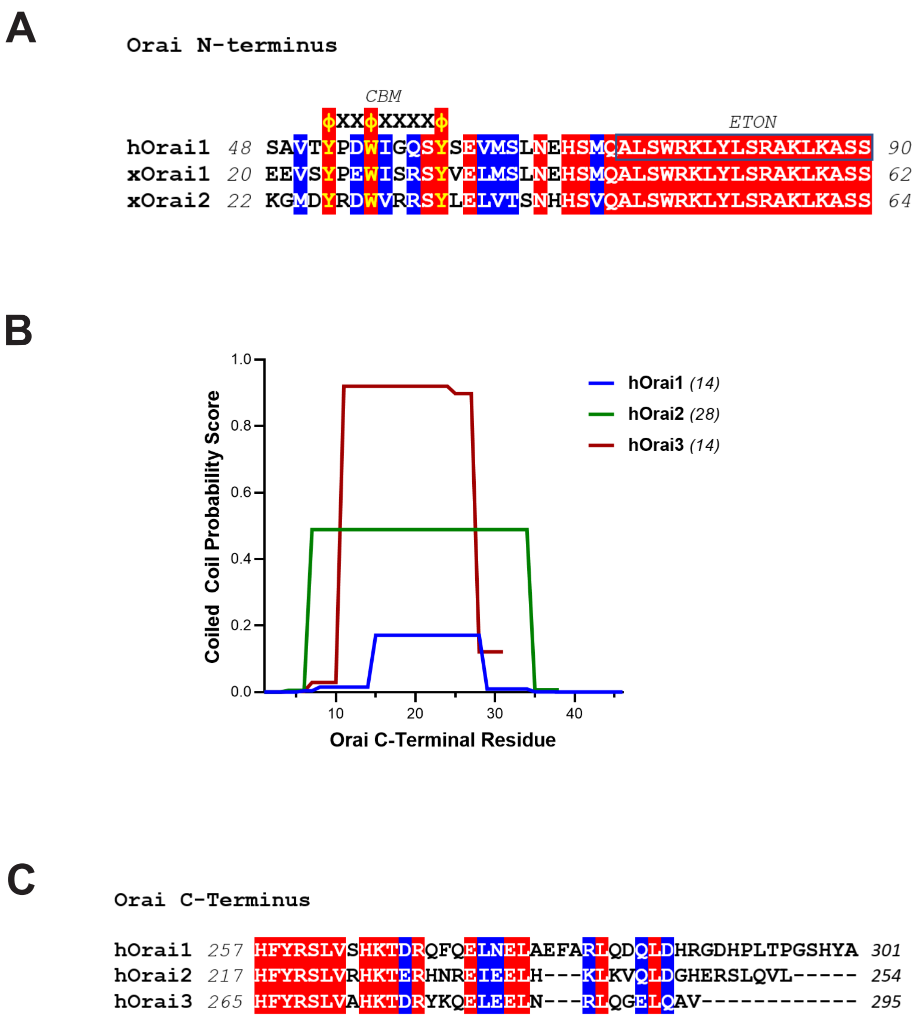

Supplement: Supplementary file 1 — Supplementary information. [file 41598_2021_82048_MOESM1_ESM.pdf]
